# Synergistic Antibacterial Proficiency of Green Bioformulated Zinc Oxide Nanoparticles with Potential Fosfomycin Synergism against Nosocomial Bacterial Pathogens

**DOI:** 10.3390/microorganisms11030645

**Published:** 2023-03-02

**Authors:** Khalid S. Almaary, Mohamed Taha Yassin, Abdallah M. Elgorban, Fatimah O. Al-Otibi, Abdulaziz A. Al-Askar, Khalid Maniah

**Affiliations:** Botany and Microbiology Department, College of Science, King Saud University, P.O. Box 2455, Riyadh 11451, Saudi Arabia

**Keywords:** drug resistance, *Hibiscus sabdariffa*, antibacterial activity, heteroresistance, fosfomycin, synergism

## Abstract

The drug resistance of bacterial pathogens causes considerable morbidity and death globally, hence there is a crucial necessity for the development of effective antibacterial medicines to address the antibacterial resistance issue. The bioprepared zinc oxide nanoparticles (ZnO-NPs) were prepared utilizing the flower extract of *Hibiscus sabdariffa* and then characterized using different physicochemical techniques. The antibacterial effectiveness of the bioprepared ZnO-NPs and their synergism with fosfomycin were evaluated using disk diffusion assay against the concerned pathogens. Transmission electron microscopy (TEM) investigation of the bioprepared ZnO-NPs showed that their average particle size was 18.93 ± 2.65 nm. *Escherichia coli* expressed the highest sensitivity to the bioinspired ZnO-NPs with a suppressive zone of 22.54 ± 1.26 nm at a concentration of 50 µg/disk, whereas the maximum synergistic effect of the bioinspired ZnO-NPs with fosfomycin was noticed against *Klebsiella pneumoniae* strain with synergism ratio of 100.29%. In conclusion, the bioinspired ZnO-NPs demonstrated significant antibacterial and synergistic efficacy with fosfomycin against the concerned nosocomial bacterial pathogens, highlighting the potential of using the ZnO NPs-fosfomycin combination for effective control of nosocomial infections in intensive care units (ICUs) and health care settings. Furthermore, the biogenic ZnO-NPs’ potential antibacterial action against food pathogens such as *Salmonella typhimurium* and *E. coli* indicates their potential usage in food packaging applications.

## 1. Introduction

A declining antibiotic research pipeline has coincided with the growth of multidrug-resistant (MDR) bacteria, which are resistant to more than three different antibiotic classes [1]. Antimicrobial-resistant (AMR) pathogens are classified as a serious danger to human health by both the World Health Organization (WHO) and the U.S. Centers for Disease Control and Prevention (CDC) [2]. According to studies, the United States experiences more than 2 million AMR infections annually, with a mortality toll of 29,000 and an associated health care cost of more than $4.7 billion [3]. Hospital-acquired (HA) and community-acquired (CA) AMR infections are responsible for about 33,000 fatalities and 874,000 disability-adjusted life years in Europe each year, costing USD 1.5 billion in direct and indirect expenses [4]. In order to focus and guide research related to the development of new antibiotics, the WHO created a list of illnesses for which the development of new antibiotics is urgently needed in February 2017 [5]. The ESKAPE pathogens (*Enterococcus faecium*, *Staphylococcus aureus*, *Klebsiella pneumoniae*, *Acinetobacter baumannii*, *Pseudomonas aeruginosa*, and *Enterobacter* species) were given “priority status” within this lengthy list [6]. ESKAPE pathogens have established resistance mechanisms against antibiotics that are the last line of defense, such as glycopeptides, carbapenems, and clinically unfavorable polymyxins, through genetic mutation and the acquisition of mobile genetic elements (MGEs) [7]. Furthermore, ESKAPE pathogens were also found to express high resistance patterns to lipopeptides, tetracyclines, macrolides, fluoroquinolones, oxazolidinones, and β-lactams antibiotics [4]. In contrast, lipoglycopeptide resistance is uncommon and has just recently been reported. This could be attributable to lipoglycopeptides’ twin function of suppressing peptidoglycan production and disrupting bacterial cell membranes [8]. Overall, the increasing proportion of bacterial species with various drug resistance mechanisms in hospital-acquired infections is due to the constitutive and/or inducible expression of these mechanisms [9].

Recent nanotechnology advancements have resulted in the development of nano-scale particles with potent bioactivity against drug-resistant bacterial infections [10,11]. For the last decade, researchers have been interested in the biological generation of ZnO NPs [12]. The prospect of using fewer chemicals, as well as its cost-effectiveness and environmental friendliness, are driving the development and importance of this green synthesis technique [13]. The biological manufacturing of ZnO NPs is undeniably more efficient than conventional chemical or physical synthesis methods [14]. Plants are the commonly used biological substrates for preparation of ZnO NPs owing to their cheap production and handling costs, minimal environmental effect, and simplicity of manufacture [15]. Furthermore, distilled water and ethanol are the most commonly utilized solvents for preparing plant extracts, and they pose fewer health concerns than microbially assisted ZnO NPs formation [16]. Plant extracts from diverse plant components, such as the leaves, bark, flowers, roots, peels, and so on, are used to make ZnO NPs [17,18]. Plant extracts are thought to contain high levels of active compounds such as flavonoids, methylxanthines, phenolic acids, and saponins. These compounds are collectively known as antioxidants [19]. These antioxidants make free radicals, reactive oxygen species, and chelated metal structures harmless. As a result, it should come as no surprise that plant extracts may serve as both bioreductors and stabilizers [20]. Plants include reductive antioxidants such as polysaccharides, polyphenols, alkaloids, tannins, amino acids, and saponins [21]. These materials also include terpenoids, alkaloids, flavonoids, and alkaloids. As a result, Zn(II) ions in corresponding salt solutions can be reduced, capped, and oxidized to form stable and well-dispersed ZnO NPs using plant extracts [22]. Several studies have used plant extracts to biosynthesize green zinc oxide nanomaterials with photocatalytic and antibacterial properties [23]. For instance, flower-like ZnO structures with hexagonal wurtzite forms were produced using Arabic Gum (AGZnO) or Karaya Gum (KGZnO) with crystallite sizes of 20 and 19 nm, respectively. Due to enhanced optical and morphological features, the KGZnO nanostructures performed better in the photodegradation of the pollutant Methylene Blue model under visible light irradiation [24]. Metal oxide nanocomposites, such as TiO_2_/Karaya nanocomposite with an average crystalline size of 10 nm generated by the sol-gel process, have been shown to photoinactivate bacterial pathogens [25]. The synergistic impact of dopants such as iron (Fe) and Praseodymium (Pr) with spherical ZnO nanoparticles may have improved their potency against *S. aureus* strain, while Pr concentration directly affected *E. coli* inhibition, making the synthesized nanomaterials promising for bacterial pathogen elimination [26]. Another previous report revealed the bioefficiency of ZnO-NPs synthesized using plantain peel extract against *Staphylococcus aureus* 26923, *Bacillus cereus* MTCC 430, *Salmonella enterica*, and *Klebsiella pneumoniae* with minimum inhibitory concentration (MIC) of 100 µg/mL against the tested strains [27]. Moreover, ZnO-NPs prepared using *Thymbra Spicata* L. extract exposed bioefficiency against the bacterial strains *Bacillus subtilis* ATCC 6633, *Escherichia coli* ATCC 25952, and *Pseudomonas aeruginosa* ATCC 27853, as well as the fungal strain *Candida albicans* ATTC 90028, with corresponding inhibitory zones of 16.3, 10.25, 13 and 10.2 mm, respectively [28]. Furthermore, a prior investigation indicated that the biologically prepared ZnO-NPs revealed antibacterial activity against *E. coli* and *Klebsiella pneumoniae* strains with minimum inhibitory concentrations (MIC) of 10 μg/mL and 40 μg/mL, respectively [29]. The previous reports have focused on evaluating the antimicrobial proficiency of ZnO-NPs and nanocomposites against different bacterial pathogens, whereas few reports have studied the synergism of ZnO-NPs with antibiotics as fosfomycin antibiotic.

*Hibiscus sabdariffa* L. (roselle) is a popular flowering plant with more than 300 species found in tropical and subtropical climates worldwide [30]. Several reports have demonstrated that the calyces of roselle are high in polyphenols and flavonoids, which increase the nutritional value of roselle since these substances are linked to antioxidant activity [31]. The plant’s phenolic content is primarily composed of anthocyanins such as delphinidin-3-glucoside, sambubioside, and cyanidine-3-sambubioside; as well as flavonoids such as gossypetine, hibiscetin, and their corresponding glycosides; protocatechuic acid, eugenol, and sterols such as β-sitoesterol and ergoesterol [32]. The high phenolic content emphasizes the potentiality of utilizing the extract as a stabilizing and reducing agent of metal ions [33]. Fosfomycin, initially known as phosphonomycin, was discovered in 1969 in Spain. Fosfomycin is available in three forms: fosfomycin calcium and fosfomycin tromethamine (a soluble salt) for oral use, and fosfomycin disodium for intravenous use [34]. Fosfomycin exhibits broad antibacterial action against both Gram-positive and Gram-negative bacteria and reveals the bactericidal activity through the inhibition of cell-wall formation by inhibiting phosphoenolpyruvate synthetase [35]. It is very effective against Gram-positive infections such as *Staphylococcus aureus* and *Enterococcus*, as well as Gram-negative bacteria such as *Klebsiella pneumoniae* and *Pseudomonas aeruginosa* [36]. Ciprofloxacin displayed a greater synergistic pattern than ampicillin, according to a previous work that examined the synergistic bioefficiency of doped ZnO-NPs with antibiotics [37]. The high incidence of antimicrobial resistance worldwide makes finding new antimicrobial combinations necessary [38]. Therefore, the current study aims to assess the antibacterial efficacy of greenly synthesized ZnO-NPs made with *Hibiscus sabdariffa* flower extract against bacteria that cause nosocomial infections, including *A. baumannii*, *E. cloacae*, *K. pneumoniae*, MRSA, and *E. coli*, as well as against the bacteria that cause food poisoning, *S. typhimurium*. Additionally, for the first time, the potential synergism between the bio-prepared ZnO-NPs and fosfomycin was examined against the tested nosocomial bacterial infections.

## 2. Materials and Methods

### 2.1. Preparation of the Plant Extract

Dried flowers of *H. sabdariffa* were obtained from a local market in Riyadh, Saudi Arabia. The Botany and Microbiology department’s herbarium confirmed the identity of the plant samples. Hisbsicus-dried flowers were cleaned three times with distilled H_2_O after being washed once with tap water. After that, they were allowed to air dry entirely. A mechanical blender was used to smash the flowers into a fine, uniform powder. A 500 mL flask containing 50 g of plant powder and 200 mL of dist. H_2_O was heated to 60 °C for 30 min. The mixture was then stirred continuously for 24 h at 25 °C using a magnetic stirrer before being cleaned using Whatman filter paper (1) to achieve a clean filtrate and eliminate any leftovers. After that, the extract was sterilized by filteration through a 0.45 µm Millipore membrane filter and a 0.2 µm Millipore membrane filter. Finally, the extracts were stored for later use in a 4 °C refrigerator.

### 2.2. Green Biopreparation of ZnO-NPs

The water extract of *H. sabdariffa* flowers was used to reduce the zinc acetate hexahydrate solution during the biopreparation of ZnO-NPs. The zinc nitrate hexahydrate salt (Zn (NO_3_)_2_.6H_2_O) of grade 98% was provided by Sigma-Aldrich, Poole, Dorset, U.K. Five mL of the flower extract was mixed with 95 mL of a 0.01 M zinc nitrate hexahydrate solution. The mixture was then stirred magnetically for an hour at 70 °C. Brown precipitate development is a sign that ZnO-NPs are developing. The reaction mixture was centrifuged at 10,000 rpm for 10 min to separate the precipitates, and the supernatant was discarded. To remove any impurities, the precipitates were subsequently rinsed with distilled water. The precipitates were then characterized after being incubated for 8 h at 100 °C in an oven.

### 2.3. Characterization of ZnO-NPs

The physicochemical characterization of ZnO-NPs was conducted using different methods. UV-VIS-NIR spectrophotometer (UV-1601, Shimadzu, Kyoto, Japan) was used to detect the surface plasmon resonance (SPR) of the bioprepared ZnO-NPs in wavelength range between 200 and 800 nm. The morphology, particle size diameter and distribution of ZnO-NPs were detected using transmission electron microscope (JEOL, JEM1011, Tokyo, Japan), whereas the elemental mapping of nanoparticles was performed using Scanning electron microscopy (SEM) coupled to an energy-dispersive X-ray (EDX) analyzer (JEOL, JSM-6380 LA, Tokyo, Japan). However, the main functional groups of ZnO-NPs were identified using Fourier transform infrared spectroscopy (Shimadzu, Kyoto, IR Affinity 1, Japan). The crystalline size and configuration were detected using X-ray powder diffraction (XRD) analysis, which was conducted using a Shimadzu XRD model 6000 diffractometer (Shimadzu, Columbia, MD, USA) fitted with a graphite monochromator. The surface charge and the average hydrodynamic diameter of the bioprepared ZnO-NPs were estimated using a Zeta sizer instrument (Malvern Instruments Ltd.; zs90, Worcestershire, UK) [39].

### 2.4. Screening of the Antibacterial Activity of the Bioinspired ZnO-NPs

The following bacterial pathogens, comprising *Salmonella typhimurium* (ATCC 14023), *Klebsiella pneumoniae* (ATCC 700603), *Enterobacter cloacae* (ATCC 13047), methicillin-resistant *Staphylococcus aureus* (ATCC 43300), *Escherichia coli* (ATCC 25922), and *Acinetobacter baumannii* (ATCC 43498), were used to determine their sensitivity to the bioprepared ZnO-NPs. Disc diffusion assay was employed to detect the antimicrobial efficacy of the bioinspired ZnO-NPs against the concerned pathogens [40]. Firstly, the microbial suspension was prepared from fresh bacterial colonies using 0.85% sterilized saline solution then the turbidity was accustomed using 0.5 McFarland standard to acquire viable cell count of 1.0 × 10^8^ CFU/mL. Afterwards, fresh Mueller–Hinton agar (MHA) plates were inoculated with 0.5 mL of the prepared suspension, which was spread homogeneously over the plates. The ZnO-NPs were suspended in methanol for disk impregnation, and then 50 and 100 μg of the solution were loaded onto sterile filter disks (8 mm), whereas the negative control disks only had methanol solvent on them. In addition, positive controls were the fosfomycin (50 μg) plus 50 μg glucose-6-phosphate. Lastly, the impregnated disks were allocated over the seeded layer and then kept in incubator for 24 h at 37 °C. MIC was estimated against *E. coli* strain, which exposed the uppermost sensitivity to ZnO-NPs using broth microdilution method as reported previously [41]. Consequently, the minimum bactericidal concentration (MBC) was estimated by plating inoculums from MIC wells onto MHA plates and incubating them at 37 °C for 24 h, with the concentration displaying no apparent bacterial growth being recorded as the MBC [42].

### 2.5. Detection of Morphological Deformations of Bacterial Cells Using SEM Analysis

Scanning electron microscope (SEM) analysis was used to observe the deformations generated by the biogenic ZnO-NPs against MRSA as a Gram-positive strain and *E. coli* as a Gram-negative strain. Small pieces of agar were removed from the inhibitory zones and fixed for 1 h at 25 °C in 3% (*v*/*v*) glutaraldehyde (buffered with 0.1 M sodium phosphate buffer, pH 7.2). Afterwards, the fixed agar pieces were rinsed four times in buffer. After the pieces had been post-fixed in 1% (*w*/*v*) osmium tetroxide (OsO_4_) for 1 h, they had been rinsed four times in buffer. Then, alcoholic dehydration of the samples was performed using a series of ethanol concentrations (30–100%) for 15 min. The samples were dried and fixed onto stubs using double-sided carbon tape. A Polaron SC 502 sputter coater then was utilized to apply a small coating of gold to the specimens. Finally, scanning electron microscope (JEOL JSM-6380 LA) was used to investigate them.

### 2.6. Detection of Synergistic Activity between ZnONPs and Fosfomycin Antibiotic

The potential synergism between the bioinspired ZnO-NPs and the fosfomycin antibiotic was evaluated by disk diffusion assay. The MIC of ZnO-NPs (30 µg) was loaded onto 8-mm disks, and then negative and positive controls were prepared. After that, the impregnated disks were placed over the surface of the inoculated plates, and then kept in incubator at 37 °C for 24 h. The synergism (%) was assessed using the succeeding formula: Synergism (%) = $\frac{T-C}{C}\times 100$, (C: referred to the clear zone of fosfomcyin and T: referred to the clear zone of the combined fosfomycin and ZnO-NPs.

### 2.7. Statistical Analysis

The statistical analysis of data was accomplished using GraphPad Prism 8.0, and the results were stated as means of triplicates ± standard error. 

## 3. Results and Discussion

### 3.1. Biosynthesis of ZnO-NPs

**Figure 1:**
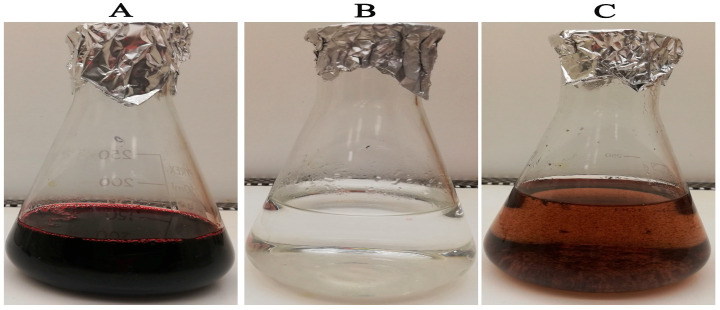
Reduction of Zn (NO_3_)_2_.6H_2_O solution using the flower extract into biogenic ZnO-NPs (**A**) *H. sabdariffa* extract, (**B**) colorless zinc nitrate solution, and (**C**) the reduced solution.

**Figure 2:**
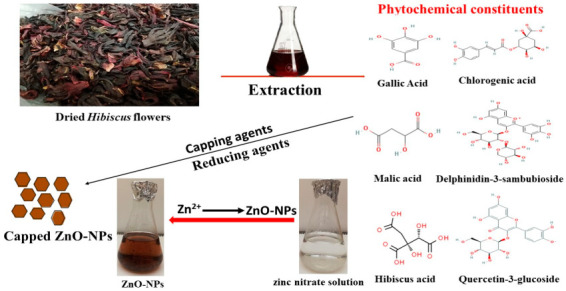
Diagrammatic illustration of ZnO-NPs biosynthesis.

Figure 1A reveals that the plant extract reduced the zinc nitrate solution resulting in formation of reduced precipitates of ZnO-NPs (Figure 1). *H. sabadriffa* flower extract was informed to be composed of polyphenols and flavonoids as gallic acid, chlorogenic acid, quercetin-3-glucoside, and protocatechuic acid [43]. Moreover, high content of anthocyanins such as delphinidin-3-sambubioside, and cyanidin-3-sambubioside were detected in the *Hibiscus* flower extract [44]. Furthermore, the water extract of *Hibiscus* contains high percentage of organic acids as citric, hydroxycitric, malic, hibiscus, and tartaric acids as major compounds, and also ascorbic and oxalic acids as minor compounds [45]. Communally, the significant amount of polyphenolic constituents, organic acids, and anthocyanins was thought to function as reducing, capping, and stabilizing agents of ZnO-NPs (Figure 2) [46].

### 3.2. UV-Vis Spectral Analysis of ZnO-NPs

**Figure 3:**
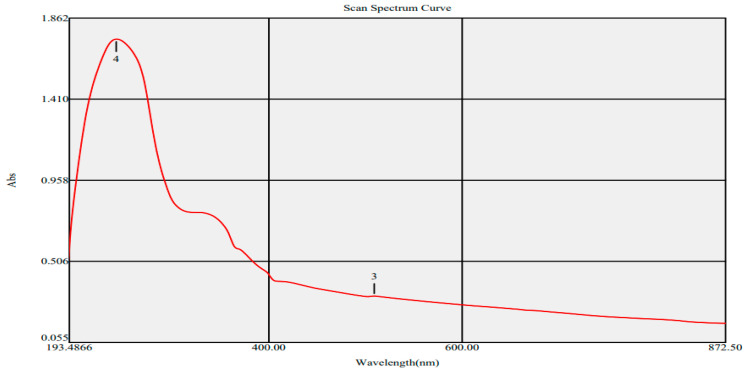
UV spectra of the bio-produced ZnO-NPs (Peak no 4: 242 nm; Peak no 3: 512 nm).

**Figure 4:**
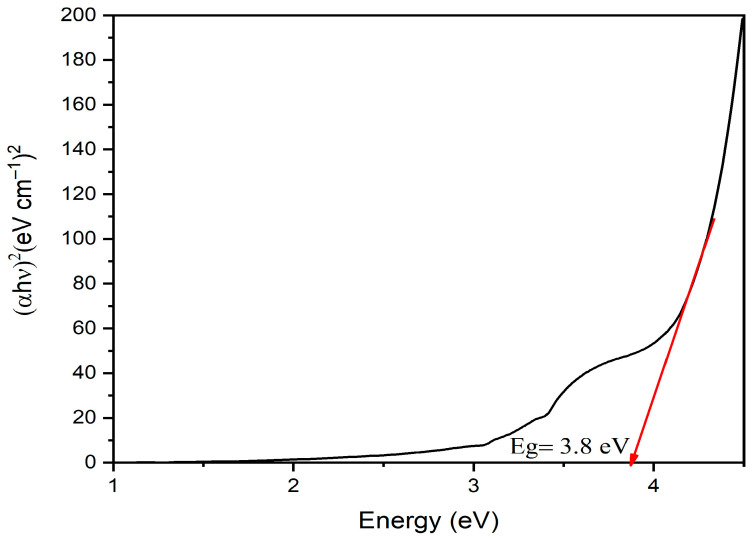
Band gap energy of the bio-produced ZnO-NPs.

UV-Vis analysis was accomplished to explore the optical characteristics of ZnO-NPs. The UV spectra of ZnO-NPs exhibited two peaks at 242 and 512 nm. The sharp band noticed at 242 nm was attributed to plant metabolites, while the wide peak detected at 509 nm was accredited to the surface plasmon resonance (SPR) of ZnO-NPs (Figure 3). Our results were in line with a prior publication that revealed pure ZnO-NPs biosynthesis using *Punica granatum* peels with an emission band at 509 nm [47]. The band gap energy of ZnO-NPs was appraised by the Tauc plot method (Figure 4). This was achieved by plotting a graph of (αhν)^2^ against hν where α = 2.303A/d, A is the absorbance, d is the thickness of the nanomaterial, h is Planck’s constant and ν is the frequency of light absorbed [48]. The band gap energy was determined to be 3.8 eV, which was consistent with prior results [49,50].

The band gap energy (3.8 eV) was greater than that of a preliminary research (3.4 eV), which reported the biopreparation of ZnO-NPs with a particle size diameter of 34 nm using *Coriandrum sativa* leaf extract. The band gap increases as particle size decreases because electron-hole pairs are now closer together and coulombic interaction between them cannot be disregarded, leading to an increase in the kinetic energy, which explains the increase in band gap energy compared to prior research [51].

### 3.3. Transmission Electron Microscope Analysis

**Figure 5:**
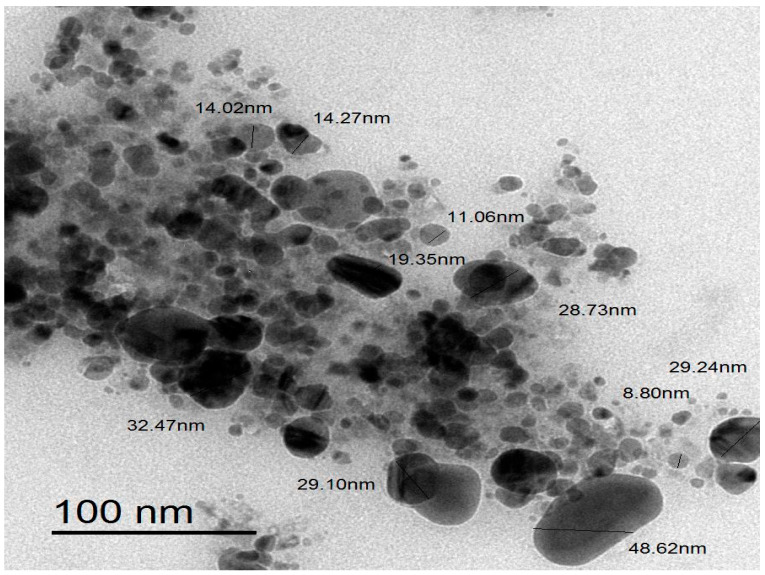
TEM graph of the bioproduced ZnO-NPs.

**Figure 6:**
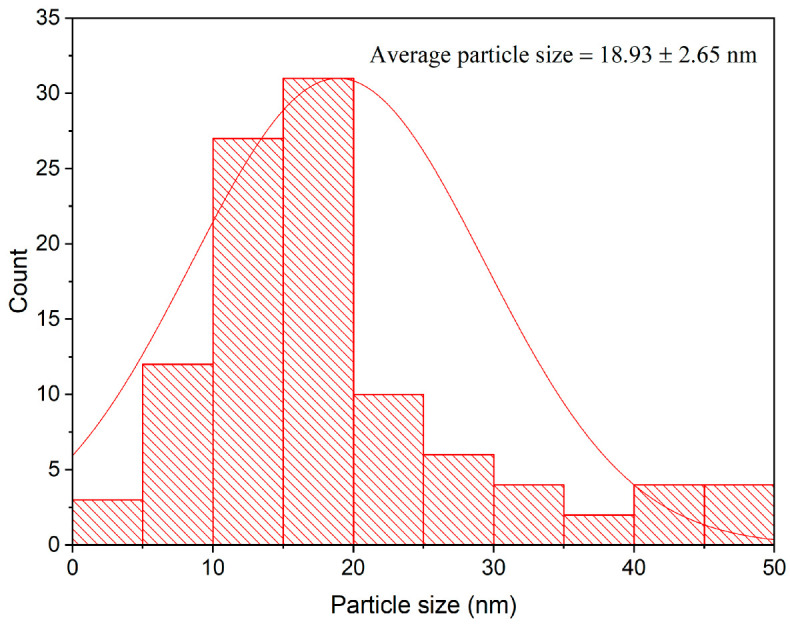
Particle size distribution histogram of ZnO-NPs.

TEM examination indicated that the bioproduced ZnO-NPs were in hexagonal form and varied in diameter from 5 to 50 nm (Figure 5). Accordingly, ZnO-NPs had an average particle diameter of 18.93 ± 2.65 nm (Figure 6). Our findings were in line with those of an earlier work that showed the biopreparation of ZnO-NPs utilizing a leaf extract from *Malva Parviflora*, with avergae size of 18 nm in diameter [52]. However, the ZnO-NPs avergae size was lesser than that of earlier studies, which showed that the ZnO-NPs prepared utilizing the leaf powder of *Ocimum tenuiflorum* and aqueous stem extracts of *Cissus quadrangularis* was 50–63 nm and 75–90 nm, respectively [53,54]. The tiny ZnO-NPs of the prepared nanoparticles demonstrated the bioefficiney of the green technique in ZnO-NPs preparation.

### 3.4. Energy-Dispersive X-ray (EDX) Analysis

**Figure 7:**
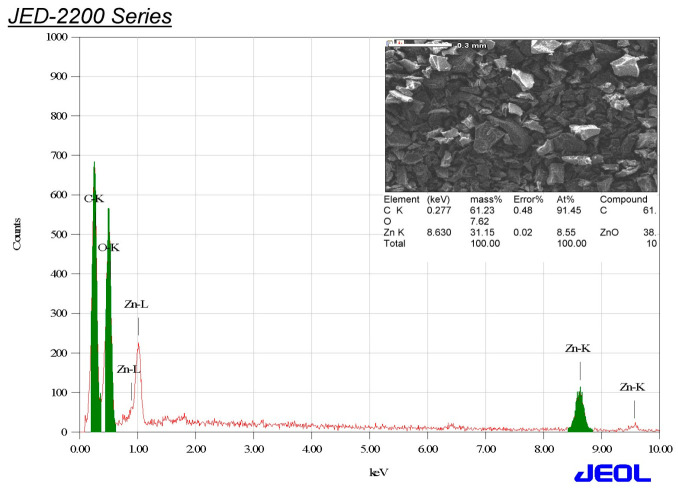
EDX spectrum of ZnO-NPs.

EDX analysis was accomplished to find out the key components of ZnO-NPs and reveal the elemental mapping of the bio-inspired ZnO-NPs. The carbon, oxygen, and zinc relative mass proportions of ZnO-NPs were determined to be 61.23, 7.62, and 31.15%, respectively (Figure 7). The stabilizing chemicals in the plant extract used functioned as capping agents for ZnO-NPs and may be the primary cause of the carbon peak [55]. Furthermore, the carbon peak might be ascribed to the carbon tape employed during sample processing [56].

### 3.5. FTIR Analysis of ZnO-NPs

**Figure 8:**
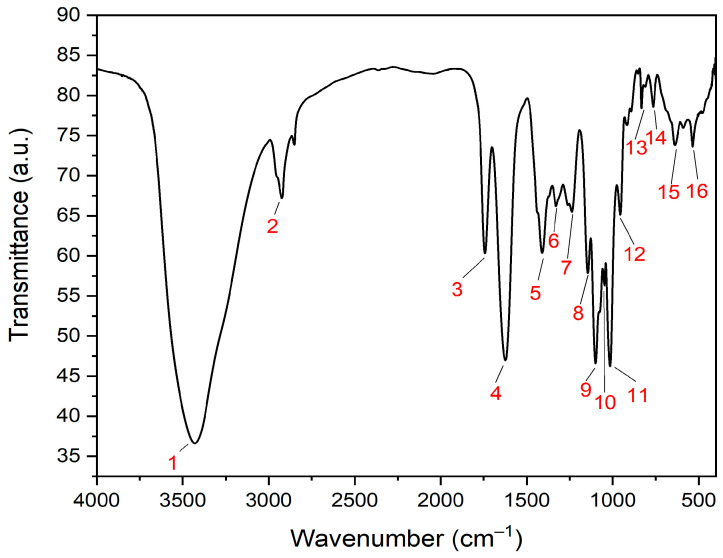
FTIR spectrum of ZnO-NPs (The numbers referred to the wavenumber of different bands (peak 1: 3429.94 cm^−1^; peak 2: 2924.56 cm^−1^; peak 3: 1742.31 cm^−1^; peak 4: 1624.74 cm^−1^; peak 5: 1410.06 cm^−1^; peak 6: 1329.53 cm^−1^; peak 7: 1237.50 cm^−1^; peak 8: 1145.03 cm^−1^; peak 9: 1100.92 cm^−1^; peak 10: 1048.07 cm^−1^; peak 11: 1014.87 cm^−1^; peak 12: 956.94 cm^−1^; peak 13: 832.32 cm^−1^; peak 14: 765.06 cm^−1^; peak 15: 638.89 cm^−1^; peak 16: 535.65 cm^−1^).

The major functional groups of ZnO-NPs that correspond to the reducing, stabilizing, and capping processes were determined using FTIR analysis. The FTIR investigation demonstrated a broad peak at 3429.94 cm^−1^, which arises from O-H group of phenolics (Figure 8). Our outcomes were in agreement with those of a previous investigation, which disclosed that *Mentha mozaffarianii* extract was employed to biosynthesize ZnO-NPs in a green way. That study also found an FTIR peak at 3429 cm^−1^, which might be accredited to phenolics’ OH bond [57]. However, the C-C stretching bonds of alcohols and carboxylic acids were ascribed to the medium FTIR band at 1410.06 cm^−1^ [58,59], while the band at 1329.53 cm^−1^ was allotted to the C-N groups of aromatic amines [60]. However, the peaks at 2924.56 and 1742.31 cm^−1^ were correlated to the C-H and C=O groups of alkanes and aldehydes, respectively [61,62]. Furthermore, the FTIR bands rises at 1624.74, 956.94, and 832.32 cm^−1^ might be ascribed to C = C groups of alkenes [63,64]. Furthermore, the medium bands noticed at 1237.50 and 1145.03 cm^−1^ were assigned to the C-N groups of amines, which were related to the protein capping molecules of the extract [65,66]. The molecular motions of C-O groups were noticed at the bands located at 1100.92, 1048.07, and 1014.87 cm^−1^, which were consigned to secondary, primary alcohols, and carboxylic acids of the extract phytoconstituents, respectively (Table 1) [67,68,69]. Furthermore, the peaks at 765.06, and 638.89 cm^−1^ were related to the C-Cl and C-Br groups of halo compounds, respectively [70]. However, the band detected at 535.65 cm^−1^ was ascribed to metal oxide bonds owing to Zn-O stretching [71].

### 3.6. XRD Analysis of the Biogenic AgNPs

**Figure 9:**
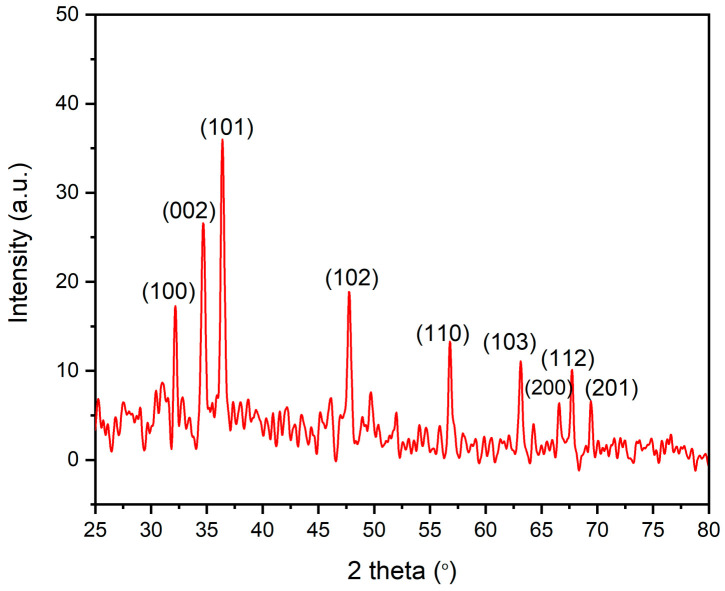
XRD pattern of ZnO-NPs.

X-ray diffraction (XRD) investigation shows the existence of nine peaks with 2θ values of 32.18°, 34.65°, 36.53°, 47.73°, 56.82°, 63.82°, 66.58°, 67.72°, and 69.41° (Figure 9) corresponding to crystal planes of (100), (002), (101), (102), (110), (103), (200), (112) and (201) correspondingly, according to the Joint Committee on Powder Diffraction Studies Standards (JCPDS card numbers 008, 82–1042 and 5–0664) [72]. These findings showed the reflection lines of hexagonal wurtzite structure of zinc oxide nanoparticles. The appearance of strong and narrow diffraction peaks in the XRD pattern verified that ZnO-NPs were crystalline in nature [73]. Scherrer’s formula was applied to estimate the crystalline size of ZnO-NPs as follow: D = (kλ/β cos θ), where D is the ZnO-NPs crystalline size, λ is the X-ray wavelength (1.54178 Å), K is Scherer’s constant (K = 0.94), θ is the diffraction angle (36.38°), β is full width at half maximum (FWHM) of the most intense diffraction peak, which was assessed to be 0.4125, and The lattice strain of the bioinspired ZnO-NPs was appraised abased on the succeeding equation: ε = $\left(\beta \right)⁄\left(4\;\tan\theta \right)$, whereas ε is the lattice strain. The assessed crystalline size was evaluated to be 21.16 nm, whereas the lattice Strain (ε) was evaluated to be 0.00548.

### 3.7. Zeta Potential Analysis

**Figure 10:**
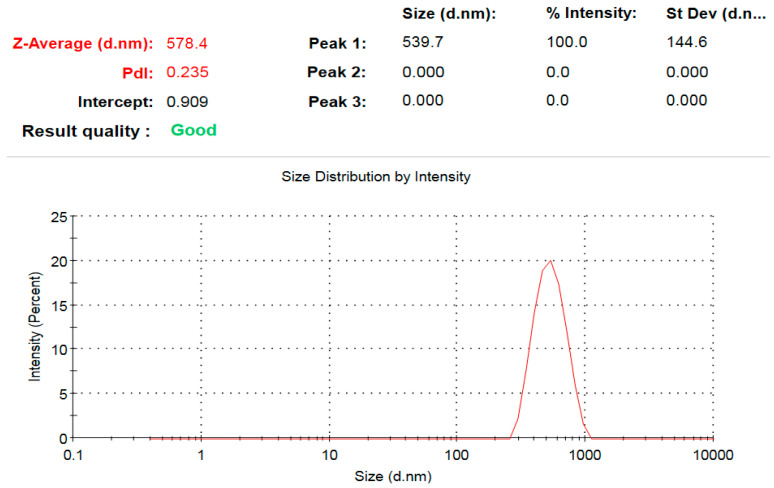
Hydrodynamic diameter of the bioprepared ZnO-NPs.

**Figure 11:**
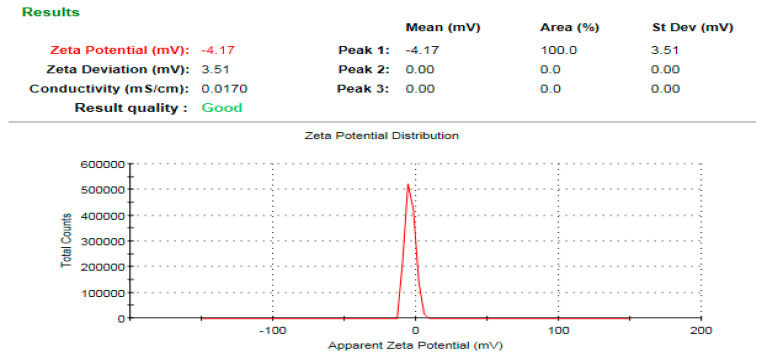
Surface charge of the bioprepared ZnO-NPs.

The hydrodynamic diameter of ZnO-NPs was assessed to be 578.4 nm (Figure 10). Because the extract capping biomolecules function as a core shell structure for the biogenic nanoparticles, the hydrodynamic diameter was greater than that determined by TEM investigation, leading to the formation of ZnO-NPs aggregates [74]. Furthermore, the large observed hydrodynamic size in comparison to the size detected by TEM micrographs may be explained by the deposition of hydrate layers on ZnO-NPs surface [42]. Zeta potential measurement shows that the negative charge of ZnO-NPs was −4.17 mV (Figure 11), and the negative charge was ascribed to the active biomolecules that were cap-sealed across the surface of the biogenic ZnO-NPs [75]. In addition, the negative charge of ZnO-NPs ensure their good colloidal stability and long term dispersivity [76].

### 3.8. Antimicrobial Proficiency of the Bio-prepared ZnO-NPs

Disk diffusion assay was performed to evaluate the antimicrobial proficiency of the bio-inspired ZnO-NPs against bacterial pathogens. Incidentally, *E. coli* revealed the maximum sensitivity to ZnO-NPs at 50 and 100 μg/disk, with estimated clear zones measuring 22.54 ± 1.26 and 25.78 ± 1.29 mm, respectively (Table 2). The *K. pneumoniae* strain displayed the lowest sensitivity to the bio-prepared ZnO-NPs at both doses, with average clear zones measuring 12.45 mm and 14.92 mm, respectively. The bioinspired ZnO-NPs (50 µg/disk) exposed antibacterial activity against *A. baumannii*, *E. cloacae* and *S. typhimurium* strains, representing relative clear zones of 14.96 ± 1.98, 17.64 ± 0.86 and 19.32 ± 1.21 mm, respectively. Our outcomes were in line with earlier research, which described the green bio-synthesis of ZnO-NPs using *Mentha mozaffarianii* extract and demonstrated a potent bioefficiency against *K. pneumoniae* strain with inhibition zones of 14 and 17 mm at ZnO-NP concentrations of 25 and 50 g/mL, respectively [57]. However, the research results contradicted those of recent research, which concluded that *K. pneumoniae* was the furthermost sensitive strain to ZnO-NPs generated using the water extract of *Ocimum americanum*, followed by *E. coli* strain, with clear zones of 33 and 31 mm, respectively [77]. *K. pneumoniae* demonstrated the lowermost sensitivity to ZnO-NPs, with a MIC value of 125 g/mL, according to a preceding investigation that confirmed the bioactivity of ZnO-NPs using *Geranium wallichianum* leaf extract. These outcome were in line with the results of the present study [78]. The biogenic ZnO-NPs revealed the uppermost antibacterial efficacy against *E. coli* strain, with an estimated clear zone measuring 25.78 ± 1.29 mm in diameter, in contrast to a previous report that concluded *E. coli* strain to be the least susceptible to ZnO-NPs, with an inhibitory zone of 4 mm, and *K. pneumoniae* to be the most sensitive, with an estimated clear zone of 31.3 mm [79]. Moreover, the biogenic ZnO-NPs demonstrated antimicrobial efficaciousness against MRSA strain with corresponding clear zones of 13.56 ± 1.34 and 15.22 ± 1.12 mm at the concentrations of 50 and 100 μg/disk, respectively. A previous study confirmed the antimicrobial efficaciousness of ZnO-NPs, synthesized using *Moringa oleifera* extract, at 10, 20 and 30 µg/disc against MRSA strain, reporting suppressive zones of 6 ± 02, 12 ± 01 and 17; ± 03 mm, respectively [80]. Altogether, the bioprepared ZnO-NPs demonstrated antibacterial bioactivity due to the effect of reactive oxygen species (ROS) generated by the biogenic ZnO-NPs [81]. The generation of ROS such as singlet oxygen (^1^O_2_) and hydroxyl radicals (HO•) results in the distraction of cellular components like lipids, proteins, and DNA, as a result of their integration into the bacteria cell membrane [82]. In addition, there is another proposed mechanism of antibacterial action through the release of Zn^2+^ ions, which have a destructive influence on the cellular active transport, amino acid metabolism, and consequently disruption of the enzymatic system [83]. Collectively, ZnO-NPs are bactericidal agents that are assimilated on the bacterial surface and thus disrupt membranes, causing membrane malfunction and leading to the internalization of ZnO-NPs into bacterial cells, which is influenced by particle size and surface chemistry, ultimately disrupting various bacterial cellular activities [84]. Intriguingly, the bioinspired ZnO-NPs have strong antibacterial activity against nosocomial and drug-resistant bacterial pathogens such as *A. baumannii*, *E. coli*, *E. cloacae*, *K. pneumoniae*, and MRSA. This suggests that these biogenic nanoparticles could be used as highly effective antimicrobial agents to control nosocomial pathogens in ICUs and other health care facilities [85]. Furthermore, the appealing antibacterial activity of bioinspired ZnO-NPs against food pathogens such as *S. typhimurium* and *E. coli* highlights potential uses of these nanoparticles in food packaging and food nanotechnology [86]. Accordingly, MIC was determined for the *E. coli* strain with the greatest sensitivity to ZnO-NPs. The ZnO-NPs had a MIC of 30 µg/mL against *E. coli* and a minimum bactericidal concentration (MBC) of 60 µg/mL. The MIC value of ZnO-NPs against *E. coli* strain, which was noticed to be 30 µg/mL, was less than that stated in a former report, which proved that the bioprepared ZnO-NPs fabricated using *Citrus sinensis* peels extract exhibited antimicrobial efficiency against *E. coli* with a MIC of 40 µg/mL [87]. The ZnO-NPs had a smaller particle size diameter of 18.93 nm in comparison with the previous study’s relative particle size diameter of 33.1 nm, which might explain why the MIC value of ZnO-NPs against *E. coli* was lower. Our results were similar with previous study, which verified that small-sized nanoparticles exhibited better antibacterial activity than large-sized nanoparticles [88]. A previous report confirmed that the small-sized nanoparticles exhibited stronger antibacterial activity due to the easy entrance of ZnO-NPs into the bacterial cell wall and also the high surface area to volume ratio, which allowed a high number of ZnO-NPs to interact directly with bacterial cell components [89]. This explains the high bioactivity of small-sized nanoparticles compared to large ones, as previously reported [90]. Subsequently, the MIC of ZnO-NPs was tested for their synergism with fosfomycin against bacterial pathogens to evaluate the minimal concentration of ZnO-NPs, which boosted the antimicrobial bioactivity of fosfomycin antibiotic.

### 3.9. Detection of the Morphological Alterations of Bacterial Pathogens

**Figure 12:**
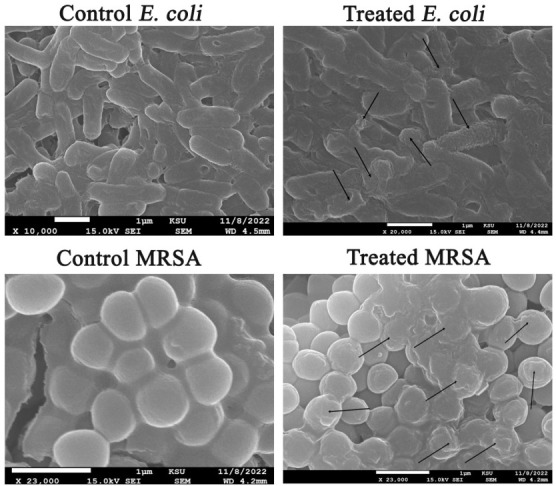
SEM graphs of *E. coli* and MRSA bacterial cells treated with ZnO-NPs (The deformations of both MRSA and *E. coli* strains were pointed out by arrows).

Scanning electron microscope was employed to inspect the morphological and ultrastructural changes in *E. coli* and MRSA cells treated with ZnO-NPs. In that regard, *E. coli* cells treated with ZnO-NPs were observed to be aberrant, damaged, malformed, and wrinkled, with a fractured outer surface and fully distorted cell membranes (Figure 12). The untreated cells were determined to be integral, normal, and rod-shaped, with high structural integrity and no observed damage. Unlike the control cells, the treated MRSA cells were observed to be distorted, with cell debris and damages.

### 3.10. Synergistic Efficiency of ZnO-NPs with Fosfomycin

**Figure 13:**
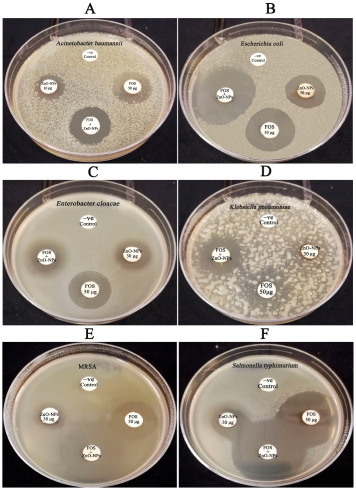
Synergistic patterns of ZnO-NPs with fosfomycin against bacterial pathogens. (**A**) *A. baumannii*, (**B**) *E. coli*, (**C**) *E. cloacae*, (**D**) *K. pneumoniae*, (**E**) MRSA, (**F**) *S. typhimurium*.

**Figure 14:**
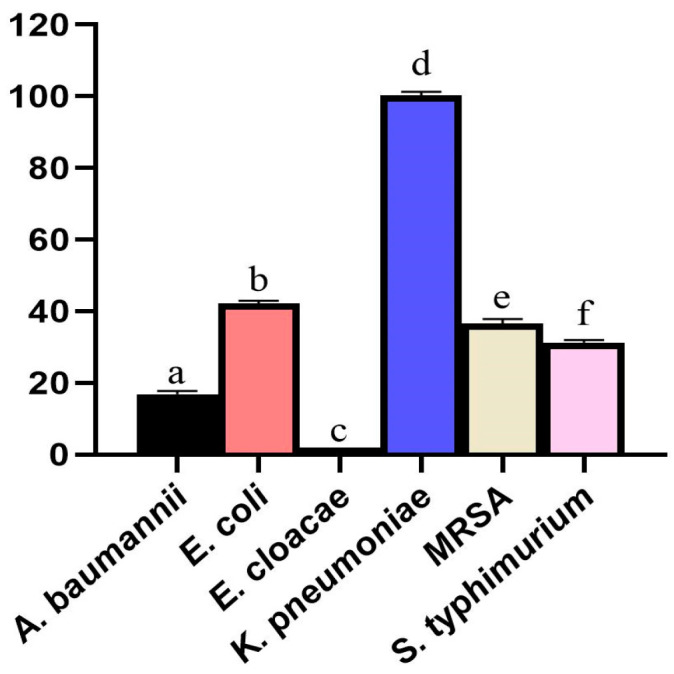
Synergistic proportions of the greenly prepared ZnO-NPs with fosfomycin against bacterial pathogens (Different letters revealed that the values were significantly different (*p ≤* 0.05)).

The disc diffusion assay was employed to examine the synergistic bioactivity of greenly generated ZnO-NPs in combination with fosfomycin against different microbes. Remarkably, the estimated clear zone of ZnO-NPs against *E. coli* was appraised to be 18.92 ± 0.69 mm, which was considerably larger than that of a former investigation, showing that ZnO-NPs synthesized using *Pinus densiflora* extracts expressed antimicrobial bioactivity against *E. coli* strain with a measured clear zone of 15 mm [91]. The measured inhibitory zones of the combined ZnO-NPs and fosfomycin were remarkably higher than fosfomycin alone, with estimated diameters measuring 36.71 ± 0.65, 32.51 ± 0.82, 31.62 ± 1.32, 23.76 ± 0.96, 20.47 ± 0.93 mm, against *S. typhimurium*, *E. coli*, MRSA, *A. baumannii*, and *K. pneumoniae* respectively (Table 3). Accordingly, the mutual bioactivity of fosfomycin and biogenic ZnO-NPs was twofold higher than fosfomycin alone against *K. pneumoniae*, with estimated synergism ratio of 100.29%, whereas antagonistic activity was recorded between ZnO-NPs and fosfomycin against *E. cloacae* strain. However, pinpoint colonies were observed in the inhibitory areas of the fosfomycin in *A. baumannii* and *E. cloacae* plates, which might be related to the fosfomycin heteroresistance (Figure 13) [92]. In summary, “heteroresistance” occurs when subpopulations of bacterial cells within a bacterial isolate exhibit higher levels of antibiotic resistance than the susceptible main population [93]. Interestingly, despite the antagonistic and weak synergistic activities of ZnO-NPs with fosfomycin against *E. cloacae* and *A. baumannii* strains, respectively, the inhibitory areas around the discs of the combined fosfomycin and ZnO-NPs were clear with no signs of the heteroresistant colonies, in a way that the ZnO-NPs enhanced the antimicrobial efficacy of fosfomycin and boosted its bactericidal efficiency. The synergistic percentages of ZnO-NPs and fosfomycin are illustrated in Figure 14. Fosfomycin was discovered and described in 1969 from several strains of *Streptomyces* spp. Fosfomycin exhibits its bactericidal action via the inactivation of the cytosolic N-acetylglucosamine enolpyruvyl transferase (MurA), blocking the production of N-acetylmuramic acid from N-acetylglucosamine, which is the initial step in the production of the bacterial wall’s peptidoglycan chain [27]. The following bacterial pathogens, MRSA, *E. coli*, and *S. typhimurium* were sensitive to fosfomycin, whereas the *K. pneumoniae* strain revealed resistance to fosfomycin antibiotic, and both *A. baumannii* and *E. cloacae* strains revealed a heteroresistance to fosfomycin. Fosfomycin principally enters virtually all sensitive bacteria through both the glycerol-3-phosphate transport system (GlpT) and the hexose phosphate transporter (UhpT) [28]. Accordingly, the loss or decreased production of these functional transporters, lower affinity to MurA, and the production of fosfomycin-modifying enzymes are all the main resistance mechanisms to fosfomycin [34].

A preceding article showed the synergistic potency of greenly synthesized ZnO-NPs with chloramphenicol, cefpirome, polymyxin B, gentamicinm, tetracycline and ampicillin antibiotics against *S. aureus* strain with relative increase in fold area of 0.89, 0.66, 0.56, 0.49, 0.29 and 0.24, respectively [94]. Other investigation confirmed the synergistic potency of ZnO-NPs with cephotaxime, ceftriaxone, cefepime, and ampicillin antibiotics against *K. pneumoniae* strain with estimated synergistic percentages of 85.71, 85.71, 57.14, and 40.1%, respectively [95]. Intriguingly, ZnO-NPs demonstrated a synergistic ratio of 100.29 percent with fosfomycin against a *K. pneumoniae* strain, highlighting the potentiality of using this combination to control nosocomial infections provoked by the resistant *K. pneumoniae* pathogen in hospitals and ICUs. An earlier work verified the synergistic potency of ceftazidime and ciprofloxacin with ZnO-NPs against the resistant clinical isolate of *A. baumannii*. This study attributed the synergistic bioefficiency to the suppression of exporting the antibacterial agent either by inhibiting efflux pumps or by increasing antibiotic entry into the cell through breaking the bacterial membrane [96]. ZnO-NPs exposed a synergistic potency with fosfomycin against the heteroresistant strains of *E. cloacae* and *A. baumannii*, indicating the boosting effect of ZnO-NPs in enhancing the fosfomycin efficiency.

The bioinspired ZnO-NPs and fosfomycin were hypothesized to exert synergistic antibacterial activity by targeting distinct cellular targets. Accordingly, fosfomycin inhibited the peptidoglycan synthesis in bacterial cell wall by inhibiting the enolpyruvyl transferase (MurA) enzyme and blocking the production of N-acetylmuramic acid from N-acetylglucosamine. The bacterial cell membrane was consequently becoming weaker, and the bioinspired ZnO-NPs’ internalization into the cell became easier. As a consequence of the bioinspired ZnO-NPs being incorporated into the bacterial cell membrane and causing membrane malfunction, the bioinspired ZnO-NPs then carried out their bactericidal activity by generating ROS, which resulted in the disturbance of crucial cellular constituents such as lipids, proteins, and DNA.

## 4. Conclusions

The bioinspired ZnO-NPs demonstrated promising antimicrobial and synergistic efficacy with fosfomycin against nosocomial pathogens, highlighting their potential applicability in the successful management of drug-resistant pathogens in hospitals and critical care units. In addition, the ZnO-NPs-fosfomycin combination could be applied for controlling the nosocomial infections in healthcare facilities and eliminating the drug-resistant pathogens. Furthermore, the bioinspired ZnO-NPs’ antibacterial ability against food microbes such as *S. typhimurium* and *E. coli* strains highlighted the potential applicability of these nano-materials in food packaging applications.

## Figures and Tables

**Table 1:** Functional groups of ZnO-NPs detected by FTIR analysis.

Peak No.	Absorption Peak (cm^−1^)	Appearance	Functional Groups	Molecular Motion
1	3429.94	Strong, broad	Alcohols and phenols	O-H stretching
2	2924.56	Medium	Alkanes	C-H stretching
3	1742.31	Medium	Aldehydes	C=O stretching
4	1624.74	Medium	Conjugated alkenes	C=C stretching
5	1410.06	Medium	Alcohols, carboxylic acid	C–C stretching
6	1329.53	Medium	Aromatic amines	C-N stretching
7	1237.50	Medium	Amines	C-N stretching
8	1145.03	Medium	Amines	C-N stretching
9	1100.92	Medium	Secondary alcohols	C-O stretching
10	1048.07	Medium	Primary alcohols	C-O stretching
11	1014.87	Medium	Esters and carboxylic acids	C-O stretching
12	956.94	Medium	Alkenes	C=C bending
13	832.32	Weak	Alkenes	C=C bending
14	765.06	Weak	Halo compound	C-Cl stretching
15	638.89	Weak	Halo compound	C-Br stretching
16	535.65	Weak	Metal oxide bonds	Zn-O stretching

**Table 2:** Antibacterial efficiency of green ZnO-NPs against bacterial pathogens.

Microbial Strains	Inhibition Zone Diameter (mm)			
	ZnO-NPs (50 μg/Disk)	ZnO-NPs (100 μg/Disk)	Fosfomycin	Negative Control
*A. baumannii*	14.96 ± 1.98	16.83 ± 1.48	20.56 ± 1.33	0.00 ± 0.00
*E. coli*	22.54 ± 1.26	25.78 ± 1.29	23.78 ± 0.97	0.00 ± 0.00
*E. cloacae*	17.64 ± 0.86	19.13 ± 1.48	20.81 ± 1.61	0.00 ± 0.00
*K. pneumoniae*	12.45 ± 1.73	14.92 ± 1.39	9.01 ± 1.02	0.00 ± 0.00
MRSA	13.56 ± 1.34	15.22 ± 1.12	22.43 ± 1.96	0.00 ± 0.00
*S. typhimurium*	19.32 ± 1.21	22.48 ± 1.34	28.36 ± 1.19	0.00 ± 0.00

**Table 3:** Inhibitory zone diameters of ZnO-NPs with fosfomycin against bacterial pathogens.

The Tested Strains	Inhibition Zone Diameter (mm)			
	Fosfomycin	ZnO-NPs (MIC)	ZnO-NPs (MIC) + Fosfomycin	Negative Control
*A. baumannii*	20.34 ± 1.07	13.81 ± 0.78	23.76 ± 0.96	0.00 ± 0.00
*E. coli*	22.87 ± 1.13	18.92 ± 0.69	32.51 ± 0.82	0.00 ± 0.00
*E. cloacae*	20.59 ± 0.78	16.31 ± 1.02	15.08 ± 1.27	0.00 ± 0.00
*K. pneumoniae*	10.22 ± 1.43	9.24 ± 0.68	20.47 ± 0.93	0.00 ± 0.00
MRSA	23.15 ± 1.08	11.83 ± 1.29	31.62 ± 1.32	0.00 ± 0.00
*S. typhimurium*	27.96 ± 0.59	15.64 ± 0.85	36.71 ± 0.65	0.00 ± 0.00

## Data Availability

Not applicable.
